# Trefoil Factor 3 (TFF3) Is Regulated by Food Intake, Improves Glucose Tolerance and Induces Mucinous Metaplasia

**DOI:** 10.1371/journal.pone.0126924

**Published:** 2015-06-17

**Authors:** Hongfei Ge, Jonitha Gardner, Xiaosu Wu, Ingrid Rulifson, Jinghong Wang, Yumei Xiong, Jingjing Ye, Edward Belouski, Ping Cao, Jie Tang, Ki Jeong Lee, Suzanne Coberly, Xinle Wu, Jamila Gupte, Lynn Miao, Li Yang, Natalie Nguyen, Bei Shan, Wen-Chen Yeh, Murielle M. Véniant, Yang Li, Helene Baribault

**Affiliations:** 1 Amgen, Metabolic Disorders, South San Francisco, California, United States of America; 2 Amgen, Metabolic Disorders, Thousand Oaks, California, United States of America; 3 Amgen, Protein Technologies, South San Francisco, California, United States of America; 4 Amgen, Lead Discovery, Thousand Oaks, California, United States of America; 5 Amgen, Pathology, South San Francisco, California, United States of America; Johns Hopkins University School of Medicine, UNITED STATES

## Abstract

Trefoil factor 3 (TFF3), also called intestinal trefoil factor or Itf, is a 59 amino acid peptide found as a homodimer predominantly along the gastrointestinal tract and in serum. TFF3 expression is elevated during gastrointestinal adenoma progression and has been shown to promote mucosal wound healing. Here we show that in contrast to other trefoil factor family members, TFF1 and TFF2, TFF3 is highly expressed in mouse duodenum, jejunum and ileum and that its expression is regulated by food intake. Overexpression of TFF3 using a recombinant adeno-associated virus (AAV) vector, or daily administration of recombinant TFF3 protein in vivo improved glucose tolerance in a diet-induced obesity mouse model. Body weight, fasting insulin, triglyceride, cholesterol and leptin levels were not affected by TFF3 treatment. Induction of mucinous metaplasia was observed in mice with AAV-mediated TFF3 overexpression, however, no such adverse histological effect was seen after the administration of recombinant TFF3 protein. Altogether these results suggest that the therapeutic potential of targeting TFF3 to treat T2D may be limited.

## Introduction

Type II diabetes (T2D) affects over 9% of the population in the United States and this incidence is expected to double or triple by year 2050 [1]. Yet despite this alarming rapid increase, much remains to be elucidated about the mechanism underlying insulin resistance and energy homeostasis (for review [2]). In general, long-term success of lifestyle modification therapy has been modest and existing pharmacological therapies and surgeries remains inadequate to treat the vast number of affected individuals.

Bariatric surgery is one of the most effective approaches to treat diabetes [3]. Within 24 hours after resection, patients fasting glucose value and insulin resistance are markedly reduced. These improvements in the metabolic profile are sustained over long periods of time along with continued body weight loss. Despite a diabetes remission rate of up to 95%, like all invasive procedures, bariatric surgery entails risks. Therefore there is considerable interest in identifying molecules which could mimic the beneficial effects of bariatric surgery. Although weight loss is one of the mechanisms by which surgery induces diabetes remission, evidence suggests that secreted factors from the foregut and hindgut may contribute to improved glucose metabolism [4].

Glucagon-like peptide-1 (GLP1) is an incretin hormone secreted by the L cells of the distal ileum in response to nutrients. In addition to stimulating insulin secretion, GLP1 attenuates post-prandial glycemia by slowing gastric emptying, inducing satiety and reducing food intake [5]. Long-acting GLP1 analogs are available to treat diabetes. While efficient, in some cases immunogenicity interferes with efficacy of GLP1 treatment over time [6], leaving an unmet medical need, and interest in identifying additional potential gut hormone candidates.

Trefoil factor 3 (TFF3), also called intestinal trefoil factor or Itf, belongs to a family of trefoil factors. All three members of the family, TFF1 and TFF2 and TFF3, are expressed along the gastrointestinal tract [7]. TFF3 is also expressed in goblet cells of the intestine, pancreatic beta cells, biliary duct epithelial cells, in oxytocin-secreting neurons from the hypothalamus, and the amygdala [8]. TFF3 is a 59 amino acid peptide. It contains seven cysteine residues, six of which form disulfide bonds to generate a clover-like structure [9]. The remaining cysteine is used to create a homodimer, the active form of the protein. No receptors for TFF3 have been identified, although it has been shown to bind to a 50 kDa intestinal membrane protein in vitro [10].

A function for TFF3 in cell growth and migration has been documented [11–13]. TFF3 promotes mucosal wound healing in mice and humans [14–16]. The level of TFF3 mRNA expression has been associated with gastroadenocarcinoma progression [17] and biliary diseases [13, 18–20]. TFF3, which maps to the Obq4 obesity quantitative trait locus (QTL), was the most significantly changed of all genes analyzed. It is transcriptionally active in beta cell and stimulates pancreatic beta cell growth in vitro [21]. In the brain, intraperitoneal (IP) injection of TFF3 peptide has been shown to improve learning and memory [22].

In an effort to identify potential intestinal secreted factors involved in glucose regulation and which could mediate the beneficial effects of gastric bypass, we analyzed the distribution and function of TFF3 in carbohydrate metabolism in vivo. The expression analysis study presented herein confirmed that TFF3 expression was distributed evenly throughout the entire length of the GI tract from the stomach to the ileum. In contrast TFF1 and TFF2 were predominantly found in the stomach and pancreas. We found that TFF3 expression in the intestine was markedly decreased in response to food intake. TFF3 overexpression mediated by an adeno-associated virus (AAV) vector in a diet-induced obesity of B6D2F1/J mice (BDF-DIO) improved glucose tolerance, without affecting body weight, fasting plasma insulin, triglyceride, cholesterol or leptin levels. Similarly, recombinant human TFF3 protein improved glucose tolerance in mice fed a 60% high fat diet. A comprehensive pathological analysis revealed that AAV-mediated overexpression of TFF3 resulted in areas of mucinous metaplasia in the stomach. In contrast, this histological abnormality was absent following recombinant TFF3 treatment, suggesting that the presence of gastric histopathological abnormalities observed and the effect of TFF3 on glucose regulation are independent events. Altogether these results suggest that the therapeutic potential of targeting TFF3 to treat T2D may be limited.

## Material and Methods

### Animals and treatments

#### Ethics statement

All animal housing conditions and research protocols were approved by the Amgen Institutional Animal Care and Use Committee. Mice were housed in a specified-pathogen free, AAALAC, Intl-accredited facility in ventilated microisolators.

Procedures and housing rooms are positively pressured and regulated on a 12:12 dark:light cycle. All animals were fed ad libitum, unless otherwise stated and received reverse-osmosis purified water ad libitum.

Eight weeks old B6.Cg-*Lep* ^*ob*^/J male mice (stock 632, The Jackson Laboratory) and C57BL/6J (stock 664, The Jackson Laboratory) were fed standard chow (2020× Teklad global soy protein-free extruded rodent diet; Harlan). Six-weeks old B6D2F1/J male mice (stock 100006; Jackson Laboratories) were group housed and fed a 60% kcal% high-fat diet (D12492, Research Diet) for six weeks.

Two days before AAV vector or protein injection, mice were divided into 3 groups based on body weight and four hours fasting glucose, measured using a drop of blood from a tail snip wound and Accu-chek active glucometers and test strips (Roche Diagnostics) [23]. For overexpression studies, AAV-TFF3 or AAV without insert (8 x 10^12^ particles per mouse in 150 μl) were injected in the tail vein of mice (IV). For recombinant protein administration, mice were injected intraperitoneally (IP) daily with human TFF3 (at 5mg/kg body weight in 0.2 ml phosphate-buffered saline (PBS) or same volume of PBS control for seven days. On the day of glucose tolerance test (GTT), mice were fasted for four hours. Administration of vehicle and recombinant proteins occurred one hour before baseline glucose measurements. At termination, mice were euthanized following AAALAC Inc. guidelines, using CO_2_ inhalation followed by a secondary method, such as exsanguination or cervical dislocation.

### Glucose tolerance test and plasma insulin, leptin, triglyceride and cholesterol measurements

Mice were fasted for four hours beginning at 6 AM on the day of the experiment. Blood samples obtained from the tail vein were used for insulin and triglyceride measurements. Following administration of glucose (2 g per kg oral gavage), glucose levels were measured immediately before and 15, 30, 60 minutes after injection. Plasma insulin content was determined by using Insulin (mouse) ultra-sensitive EIA kit (80-INSMSU-E10, ALPCO Diagnostics).

At termination, mice were euthanized, the blood was collected by cardiac puncture, and various tissues were harvested for histological analysis. Plasma triglyceride levels were measured by using Infinity Triglyceride Reagent (TR22321, Thermo Scientific). Serum leptin levels were measured using Mouse/Rat Leptin ELISA kit, Ref# RD291001200R.

### Expression analysis

TFF3 protein levels were determined by ELISA using the anti-TFF3 from R&D Systems (catalog # AF4407).

RNA expression levels in various tissues were measured by qPCR. Briefly, the entire GI tract was removed from mice. Sections of the duodenum, jejunum, ileum and colon were cut. Food content was first squeezed out of the lumen. After a longitudinal cut, with the lumen exposed, tissue was quickly rinsed in PBS. Total RNA from brain, heart, kidney, liver, lung, pancreas, skeletal muscle, spleen and testis were obtained from Clontech and treated with DNase (Promega M6101) to remove traces of genomic DNA purified with the RNeasy micro system (Qiagen Catalog: 74004). QPCR was performed on a 7500 Fast system Applied Biosystem; all the PCR reagents were obtained from Applied Biosystems, the one-step qPCR kit (Catalog: 11732–020) was used following the manufacturer’s instructions. All samples were analyzed in duplicate at least and corrected for mouse GAPDH (BC083149) run as an internal standard. Mouse TFF primers were ordered from Applies Biosystems (TFF1 Mm00436945_m1, TFF2 Mm00447491_m1, TFF3 Mm00495590_m1).

### Recombinant protein

Human TFF3 recombinant protein was produced in a yeast expression system similarly to Wang et al. [20] with the following modifications. An N-terminal glycine was added to enhance efficient cleavage from the yeast alpha factor signal peptide of pPICZα and expression was 24 hours instead of 48 hours. Two liters Pichia pastoris cell culture expressing human TFF3 were diluted 4X by 20 mM Tris, pH 8.0. The diluted conditioned medium was collected and loaded on an 80 mL Bed Volume anion exchange Q column at 8 mL/min. The flow through (FT) was collected and acidified to pH 2.5 with HCl while stirring. The material was then loaded on a 60 mL bed volume preparative C4 reversed phase chromatography (Jupiter, 5 μm) column. Buffer A: 0.1% TFA in H2O, Buffer B: 0.1% TFA in 100% ACN. Bound TFF3 was collected from an elution gradient of 5–55% buffer B. The C4 pool of TFF3 was further polished and cleaned of Endotoxin by a 50mL SourceQ chromatography. Buffer A: 20 mM Tris, pH 7.4; Buffer B: 20 mM Tris, pH 7.4, 1M NaCl. The bound TFF3 was eluted and collected with a gradient of 5–30% B. The sample was lyophilized at -55°C for 24 hours and re-suspended in 20 mM Tris, pH 7.5, 135 mM NaCl, and stored at -80°C. Protein samples were analyzed by sodium dodecyl sulfate–polycrylamide gel electrophoresis (SDS–PAGE) using 15% acrylamide. Gels were stained with SimpleBlue (Invitrogen) and de-stained with water.

### Histopathology

Tissues were fixed in 10% Neutral Buffered Formalin for 24 hours, processed to paraffin blocks, cut into four μm sections and stained with hematoxylin/eosin according to standard procedures.

### In vitro wound healing assay

Prior to plating cells, a grid was drawn on the bottom of 6-well plates to indicate a starting point for the scratch wound response. Next, IEC-18 cells (ATCC, CRL-1589) were seeded onto 6-well plates at a density of 1.5x10^6^ in complete culture media containing DMEM (ATCC, 2002–30), 5% FBS, Penicillin-Streptomycin (P/S), and 0.1mg/ml of bovine insulin (Sigma, I0516). Upon confluence, cells were gently rinsed and cultured overnight in “starvation” media containing DMEM, P/S, and 0.1% fatty acid-free BSA (Sigma, A7030). To begin the assay, a sterile cell scraper was used to “scratch” the bottom of the well clean from the “start” site to the right side of the well. Wells were rinsed twice with starvation media to clear debris. Media containing up to 20 nM TFF3 or complete media was added to each well as indicated. Images of the wells were captured to record the start sites, and the cells were incubated at 37°C for 18 hours. At the end of the incubation time the wells were imaged again to record the migration “stop” sites. A ruler was used to determine the length of the wound response.

## Results

### TFF3 expression along the small intestine is regulated by food intake

**Fig 1:**
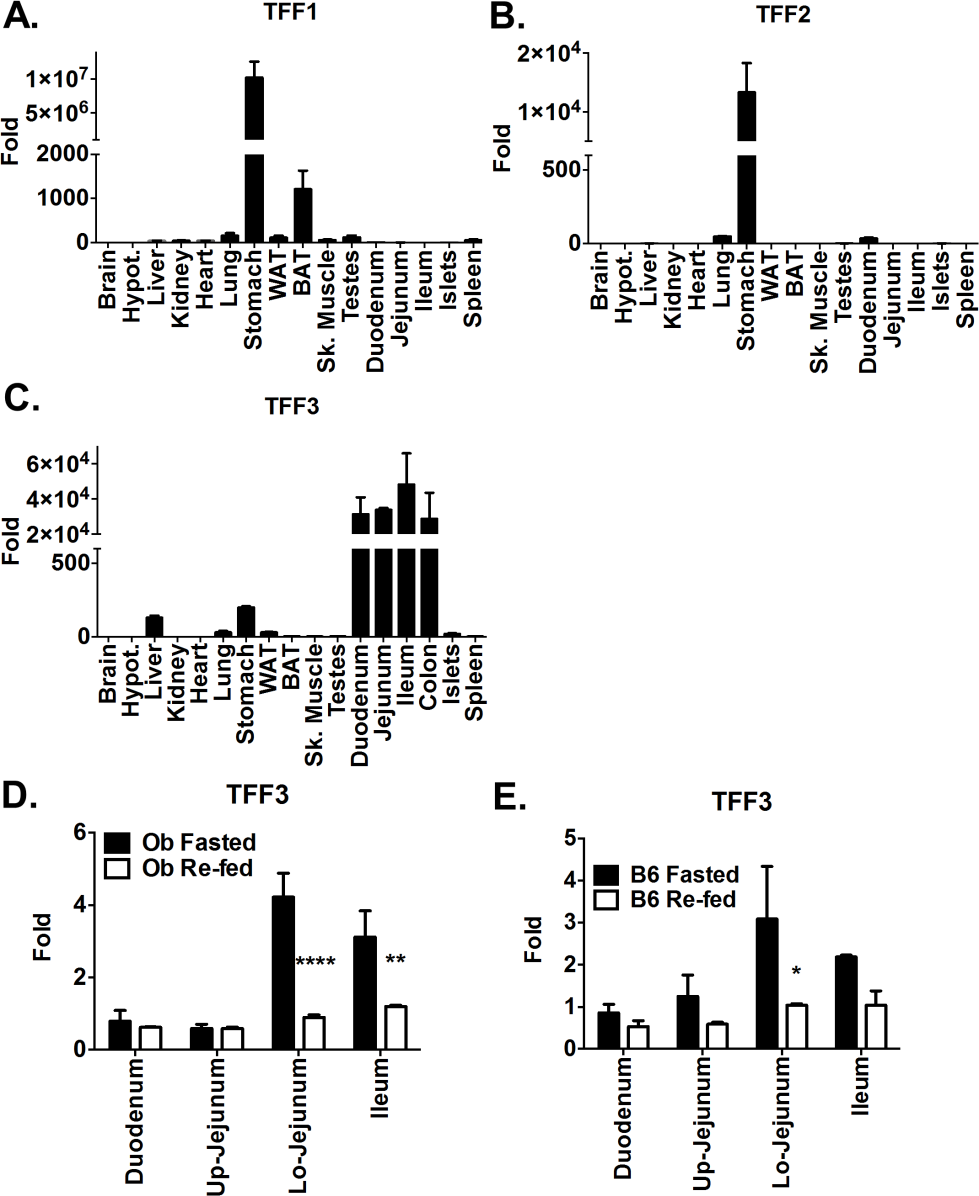
Expression of trefoil factor family members in various mouse tissues. (A-C) mRNA levels of (A) TFF1, (B) TFF2, (C) TFF3 in various organs as indicated. Values presented are fold change relative to the brain level of expression (D-E) Effect of four hours feeding ad libitum on TFF3 mRNA expression levels in the gastrointestinal tract of B6.Cg-*Lep* ^*ob*^/J (D) or C57BL\6J (E) mice previously fasted overnight. Up. And Lo. indicate the upper and lower part of the jejunum respectively (D, E) Values are presented as fold change relative to the fasted duodenum level. Error bars represent +/- S.E.M. P values after two-way ANOVA are indicated as follows: * p < 0.05, ** p < 0.01, **** p< 0.0001.

Expression of trefoil factors was analyzed in various organs (Fig 1). All three factors were present in mouse stomach, although levels of TFF1 and TFF2 were markedly greater than that of TFF3. A low level of TFF1 expression was also found in brown adipose tissue. TFF3 was predominantly expressed in all sections of the small and large intestine. Low levels of TFF3 were also found in the stomach and liver.

Because of TFF3 expression in the small intestine, we tested whether TFF3 is regulated by food intake. B6.Cg-*Lep* ^*ob*^/J mice were fasted overnight to measure the level of TFF3 mRNA in a mostly empty GI tract (Fig 1D). Half of the mice (n = 4) were then fed for four hours prior to tissue collection while the other half were kept in fasting conditions until tissue collection. Levels of TFF3 mRNA expression in fasting animals were markedly higher in the lower part of the jejunum and in the ileum. Re-feeding the mice for four hours reduced the expression in all sections, and most prominently in the lower jejunum and ileum. Similar high levels of TFF3 were seen in the lower jejunum of C57BL/6J mice (Fig 1E). Upon re-feeding these levels decreased in the lower jejunum and to a lesser extent in the remaining sections.

### Overexpression of TFF3 improves glucose tolerance

**Fig 2:**
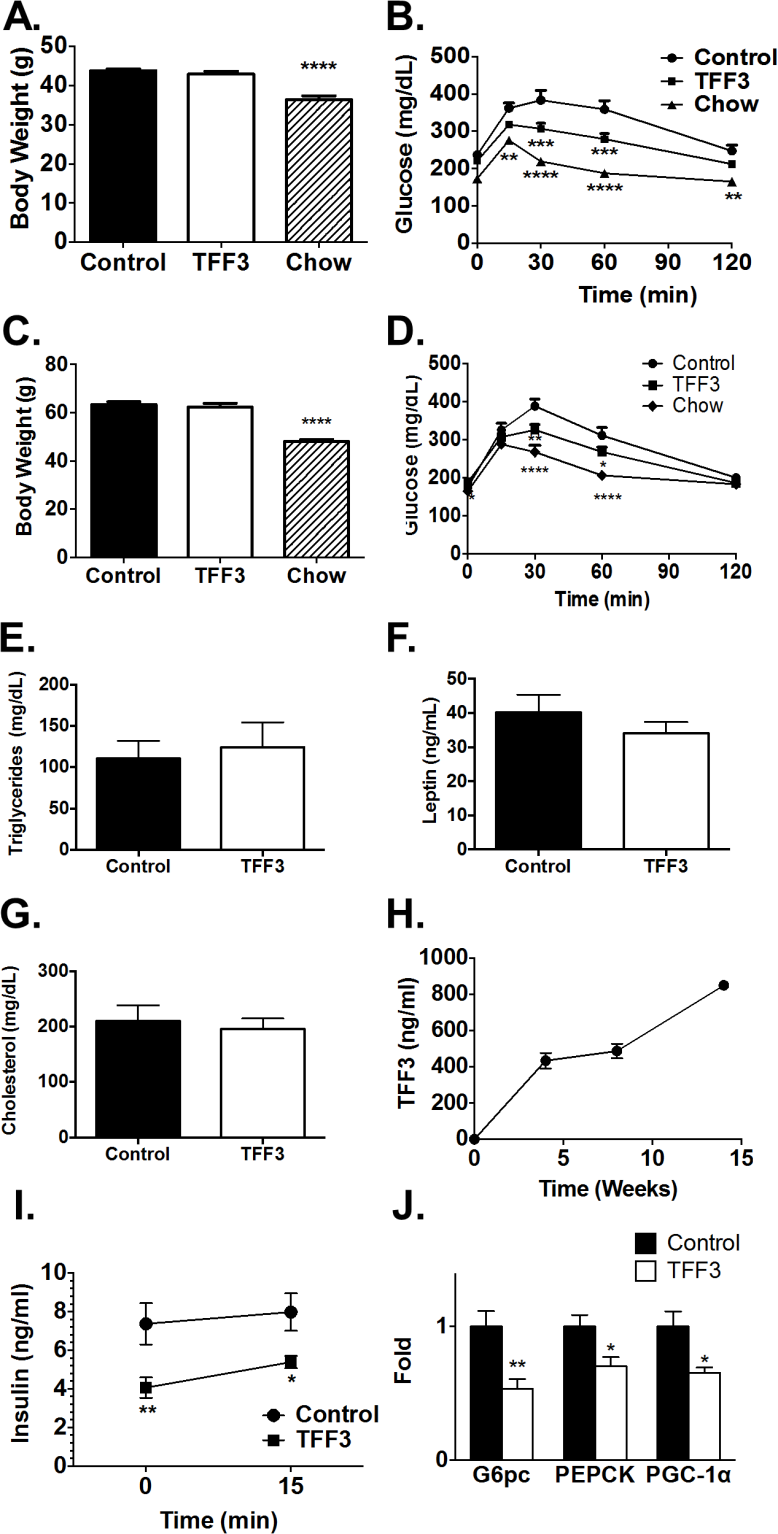
The effect of AAV-mediated TFF3 overexpression on the metabolic profile in BDF-DIO mice. B6D2F1 mice were maintained on high fat diet and were injected with an AAV vector containing human TFF3 cDNA (TFF3) or without inserted cDNA (Control) (n = 15/group). An additional group was returned to a standard chow diet (n = 8). Body weight (A) was measured and an oral glucose tolerance test was performed (B) four weeks after AAV injection. At 14 weeks after injection, six mice from each control and TFF3 treated group were euthanized. and total triglycerides (E), cholesterol (F), leptin (G) were measured. At 25 weeks after injection, the same lack of effect of TFF3 on body weight (C) and a small but significant beneficial effect of TFF3 on glucose tolerance (D) was observed in the remaining mice (Control n = 9, TFF3 n = 9, Chow n = 8). Serum level of TFF3 at various times after injection was determined by ELISA (H). Glucose-induced insulin secretion was measured two weeks after AAV-TFF3 injection (I). Expressions of G6pc, PEPCK and PGC-1α in mouse liver two weeks after TFF3 or control AAV injection (J). Error bars represent +/- S.E.M. One-way ANOVA was performed for all graphs, except for (C) and (I), for which two-way ANOVA was performed. **p<0.01, ***p<0.001 and ****p<0.0001.

B6D2F1 are F1 hybrid between C57BL/6J and DBA2/J mice known to be highly responsive to a high fat diet as a model of diet-induced obesity [24]. In addition, the low level of aggressive behavior in B6D2F1 males allow for easy randomization and regrouping of males in cages per treatment group. To test whether the overexpression of TFF3 can protect rodents from developing a diabetic phenotype, B6D2F1 mice fed a high fat diet for 6 weeks (BDF-DIO) were injected with adeno-associated viruses (AAV) containing a cDNA for human TFF3 (TFF3) or AAV vector without a cDNA (Control) (Fig 2). A third group of mice (Chow) were returned to a standard chow diet, instead of being injected. This served as a positive control as this treatment reduced body weight (Fig 2A) and improved glucose tolerance (Fig 2B). TFF3-treated mice showed significantly improved glucose tolerance (Fig 2B), albeit to a lower extent than the effect obtained by the reversal to a lean diet. In contrast, body weight was unaffected by TFF3 compared to control mice, The effect of TFF3 on body weight and glucose metabolism was seen as early as nine days after injection and lasted for more up to 25 weeks after injection (Fig 2C and 2D). No significant differences were observed in serum triglyceride (Fig 2E), leptin (Fig 2F), and total cholesterol (Fig 2G) levels from terminal blood samples. Fasting insulin levels were reduced in TFF3-treated animals. Glucose-induced insulin secretion was not significantly different in TFF3-treated and control animals (Fig 2I).

We confirmed that TFF3 was expressed at high levels, from 400 to 800 ng/ml over the first 14 weeks after injection, by performing ELISA using an anti-human TFF3 antibody (Fig 2H.) The absence of initial signal confirms that the TFF3 antibody detects the transgenic form of TFF3 and not the endogenous mouse TFF3. This level was well above commonly observed physiological serum TFF3 levels which range in human from 5 to 20 ng/ml, even in disease state such as in patients with gastric cancer [25]

We measured the expression of gluconeogenic genes by real-time PCR in the livers of TFF3-treated and control mice. The mRNA levels of G6pc, PEPCK and PGC-1α were all downregulated (Fig 2J).

### Recombinant human TFF3 improves glucose tolerance in mice

**Fig 3:**
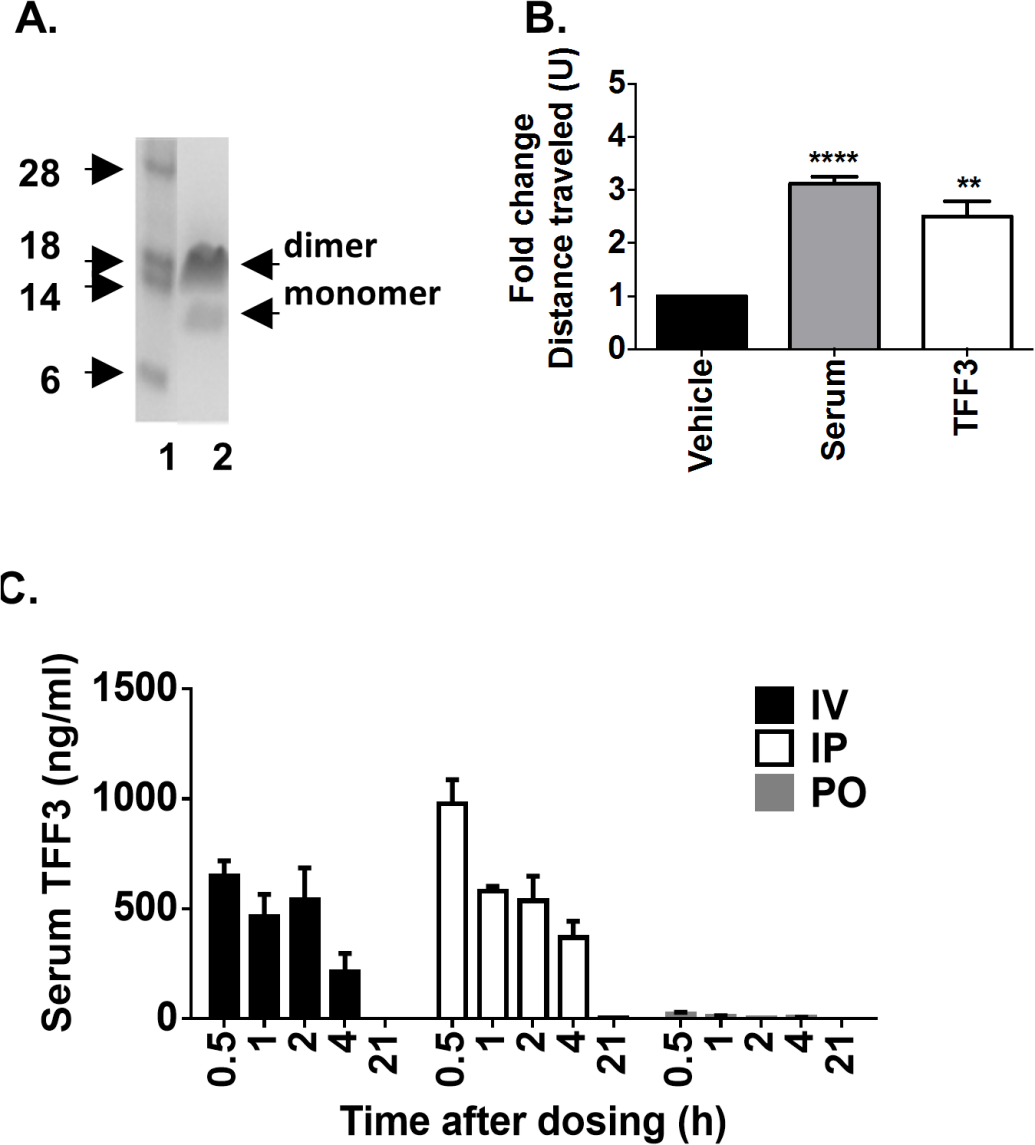
Production and characterization of recombinant TFF3 protein. (A) SDS-PAGE showing M.W. markers (lane 1) and recombinant TFF3 (lane 2). The 14 kDa band represents a TFF3 dimer and the 7 KDa band a monomer. (B) TFF3 protein was tested in an in vitro wound healing assay and compared to cells receiving vehicle alone or serum-supplemented medium. Results are shown as fold of migration distance when compared to treatment with vehicle only. (C) Pharmacokinetic properties of recombinant human TFF3 after administration in BDF-DIO mice intravenously, IV, intraperitoneally, IP, or per oral gavage, PO. **p<0.01 and ****p<0.0001.

We produced recombinant TFF3 protein using a yeast expression system. The resulting eluate contained a major band migrating at 14 kDa and a minor band at 7 kDa as observed under non-denaturing conditions (Fig 3A). Under denaturing conditions, one major band migrating at 7 kDa was identified (data not shown). Given that the predicted molecular weight of TFF3 is 7 kDa, these two bands likely represent a covalently bound dimer and a monomeric forms of TFF3.

We confirmed that recombinant TFF3 was functional using an in vitro wound healing assay. A confluent IEC-18 cell monolayer was wounded with a scalpel creating an approximate 5 mm gap and cells were allowed to grow overnight in either defined media, serum-supplemented media, or defined media supplemented only with recombinant TFF3 (Fig 3B). Cells cultured in the presence of TFF3 were able to repair the in vitro wound similarly to cells cultured in the presence of serum, thus confirming that purified recombinant TFF3 produced was active.

The stability of the recombinant TFF3 was tested in vivo (Fig 3C). TFF3 protein was administered to BDF-DIO mice either IV, IP or per oral gavage (PO) and levels of serum TFF3 was measured at various times after dosing (Fig 3D). Approximately half of the initial amount of protein found at 30 minutes was still present two to four hours after IV or IP injections. Serum TFF3 was detectable after PO dosing, albeit at a low level, possibly due to the intestinal epithelium barrier or alternatively due to rapid protein degradation in the gut. Based on these results we designed an in vivo functional study implementing daily IP TFF3 injections within one hour of the dark cycle when mouse activity, including feeding, is highest.

**Fig 4:**
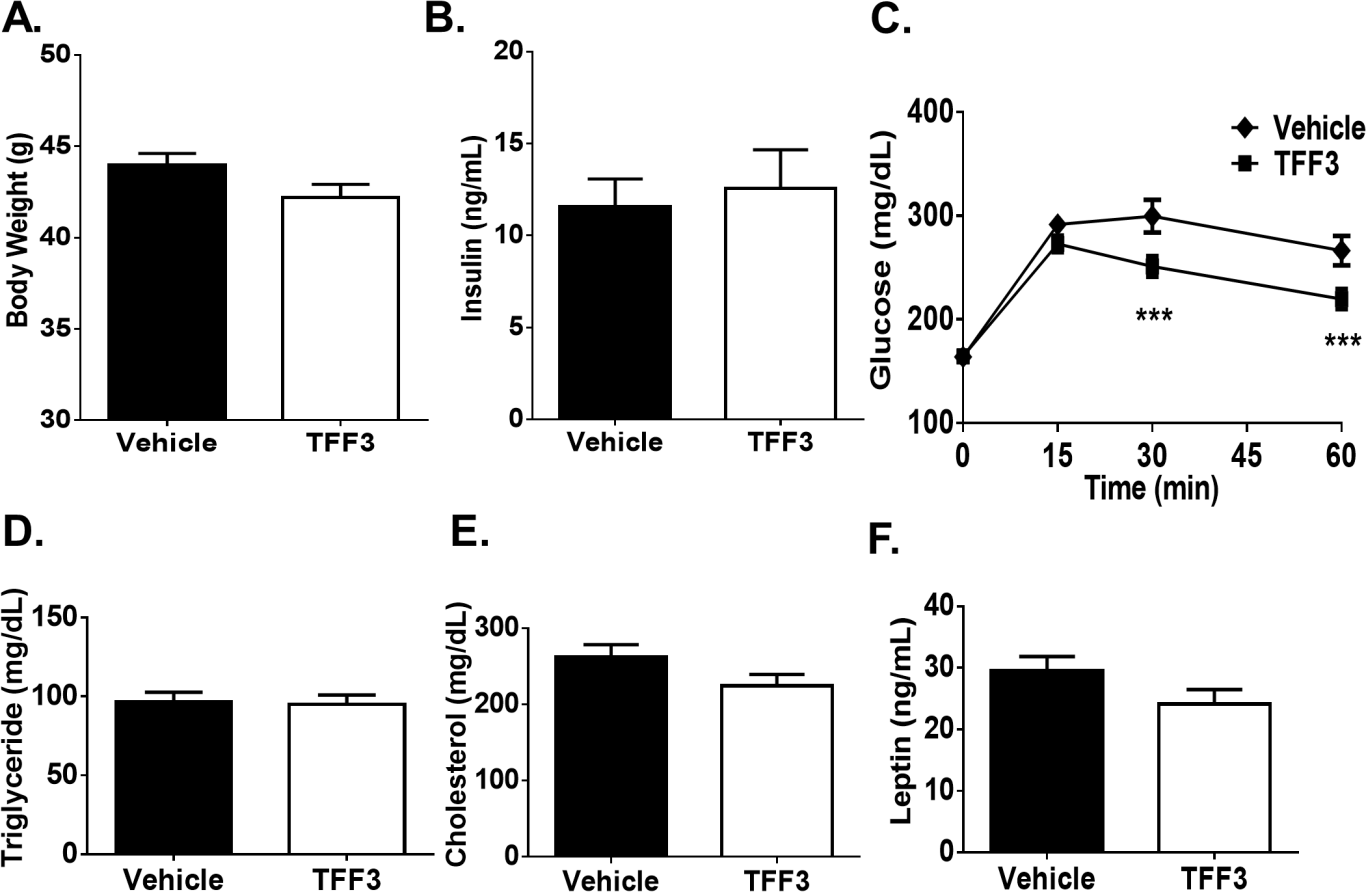
Effect of TFF3 protein on glucose metabolism. Recombinant TFF3 or vehicle were injected IP daily in BDF-DIO mice (n = 12 per group). After seven days, body weight (A) and fasting insulin (B) were measured and an oral glucose tolerance test (C) was performed. Serum triglycerides (D), cholesterol (E) and leptin (F) were measured one week later upon study termination. Error bars represent +/- S.E.M. One-way ANOVA was performed for all graphs, except for 2C for which two-way ANOVA was performed. ***p<0.001.

Daily injection of 5 mg/kg of TFF3 for seven days resulted in improved glucose tolerance (Fig 4). Similar to that observed with TFF3 overexpression, recombinant TFF3 failed to induce a significant change in body weight, fasting serum insulin and glucose levels and in serum levels of triglyceride, total cholesterol and leptin. (Fig 4).

### Overexpression of TFF3 results in mucinous metaplasia

**Fig 5:**
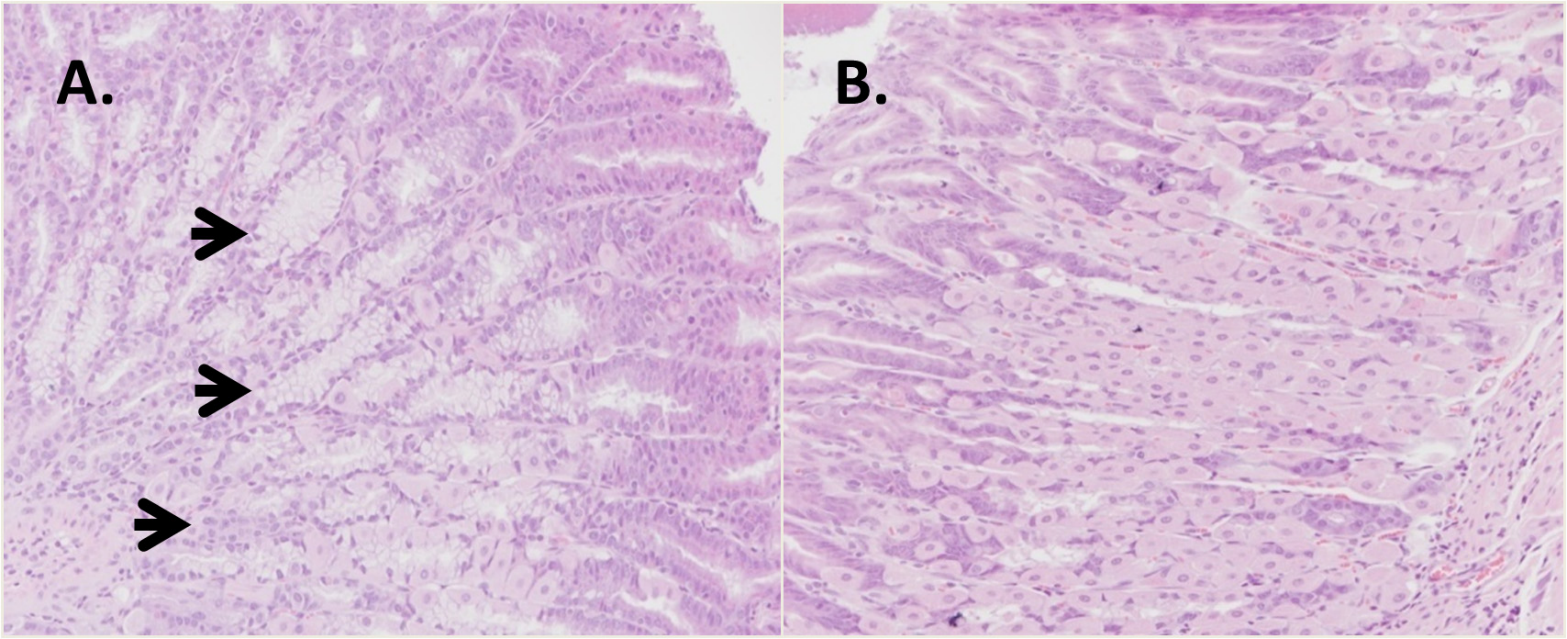
Effect of overexpression of TFF3 on the stomach histology. Stomach tissue from mice that were injected with AAV-TFF3 (A) or the control vector (B) were collected 14 weeks after injection. Four out of six TFF3-treated mice showed clear areas of mucinous metaplasia of the fundic glands, while none of the control group showed this change. All samples were stained with hematoxylin-eosin. Arrows indicate regions of mucinous metaplasia and neutrophil infiltration. All magnifications 100X.

Pathology analysis was performed at termination of the studies to determine whether the overexpression of TFF3 revealed potential safety liabilities. We found that four of the six mice analyzed in the AAV-TFF3 group showed mucinous metaplasia of fundic glands in the stomach (Fig 5 and Table 1). None of the AAV control group showed any metaplastic changes. Similar changes have been reported in mice after chemical or infectious damage to the gastric mucosa [26]. However, chemical or infectious damage is an unlikely cause of the metaplasia after TFF3 treatment in the present studies, as both groups were exposed to the same environment and originated from the same source. Animal health monitoring also showed no presence of potential infectious agents. No significant histological abnormalities were seen in mice injected with recombinant TFF3. Whether the difference observed between the AAV-mediated delivery of TFF3 and IP injection of the recombinant TFF3 protein was due to the route of administration or the length and levels of exposure remains to be determined.

**Table 1:** Presence of mucinous metaplasia.

	Control	TFF3
**AAV**	**0/6**	**4/6**
**Recombinant Protein**	**0/12**	**0/12**

Mice were injected with an AAV vector expressing TFF3 or a control empty vector, or mice were treated with recombinant TFF3 protein or a vehicle control. The entire stomach was collected, cut longitudinally, laid flat on a piece of cardboard then embedded for histological analysis of the entire length of the stomach.

## Discussion

The studies presented here show that the expression of TFF3 mRNA along the gastrointestinal axis is down-regulated in response to food intake in two different mouse models. Interestingly, a small but significant decrease in TFF3 serum level was also observed in humans in a cohort of healthy volunteers after food intake [27]. The expression of TFF1 and TFF2 is mostly restricted to the stomach. In contrast, the gastric level of TFF3 is relatively low. Rather, TFF3 expression is abundant along the entire length of the small intestine and the colon. This spatial distribution of TFF3 and the response of TFF3 to nutrients led us to investigate the role of this peptide in glucose metabolism.

We found that increased levels of TFF3 improved glucose tolerance in a diet-induced obesity mouse model. These findings are consistent with several reports that TFF3 plays a role in energy metabolism. For example, TFF3 has been shown to stimulate beta cells proliferation in human and rodent islets while preserving their functions [21]. TFF3 has also been reported to be expressed in the bile ducts of normal human liver and is upregulated in diseased livers [18]. Liver TFF3 expression levels correlate with increased glucose tolerance in Tally-Ho mice, a multigenic moderately obese mouse model of T2D [28]. Consistently, TFF3 expression was identified as having the highest fold-change in the liver of a congenic mouse strain with segments of CAST/Ei DNA on a C57BL/6J background, selected for high nutrient intake per gram of body weight [29]. TFF3 also localizes on the Obq4 obesity QTL locus resulting from an AKR/J and C57L/J intercross [30].

Recently, Xue et al. [31] reported that, using an adenovirus expression system, overexpression of TFF3, inhibited the expression of gluconeogenic genes and improved glucose tolerance and insulin sensitivity. In agreement with our observations, TFF3 overexpression showed no significant effect on body weight. Given our findings and those of Xue et al. the observation that TFF3 knockout mice have a reduced body weight is unexpected [32]. TFF3 deficiency also weakens the defense of intestinal mucosa, raising the question of whether the published body weight loss in the TFF3-KO mice is caused by a change in energy homeostasis or is a consequence of increased sensitivity to inflammation [33].

The molecular mechanisms underlying the beneficial effect of TFF3 on glucose metabolism remain unclear. When administered over a long period with AAV, TFF3 beneficial effect was found associated with the presence of mucinous metaplasia. However, this association was absent from our studies with the recombinant TFF3 protein suggesting that the gastric pathological defect and improvement on glucose tolerance are independent events. While TFF3 is predominantly expressed in the GI tract, expression is also found at lower levels in other metabolically relevant tissues, notably pancreatic islets, liver and gallbladder. Therefore TFF3 may act via multiple organs. Although TFF2 has a different distribution along the GI tract than TFF3, genetic ablation of TFF2 confers resistance to diet-induced obesity [34] corroborating the role of TFF family peptides interact in metabolic regulation.

To date, a receptor for TFF family members remain to be identified. Binding affinities of TFF3 to intestinal plasma membranes suggest strongly that such a receptor exists [35]. A 50 kDa candidate protein from an intestinal membrane fraction was identified using a ligand blotting technique, although further studies are needed to demonstrate that this binding protein bears receptor activities [10]. Similarly TFF2-binding molecules were identified, such as CRP-ductin and a fibronectin receptor β subunit, but further studies are needed to verify whether these molecules are TFF receptors [36].

A role for TFF3 has been suggested in a large range of physiological functions. TFF3 can reduce adhesion between cells and thereby promote migration and mucosal repair [9, 11, 13, 37]. We have used an in vitro wound healing assay to demonstrate the activity of recombinant TFF3. We confirmed that our purified protein could promote wound closing at a level nearing serum-supplemented medium. Increased migratory activity is often associated with increased neoplasia risk and metastatic behavior. In line with this, elevated levels of TFF3 are seen in prostrate, colon and gastric cancers [9, 17, 38–43]. Promoter polymorphisms in the TFF3 gene are associated with diffuse-type gastric cancer in a population of Chinese males [44]. Circulating TFF3 levels are dramatically elevated in chronic kidney disease [45], which in turn is correlated with an elevated risk to develop cancers [46].

In the studies presented here, we observed that TFF3 overexpression causes mucinous metaplasia. Intestinal mucinous metaplasia is defined by the change in appearance of parietal or chief cells in the stomach to an intestinal like cell containing mucin. In mouse models, the incomplete differentiation into differentiated enterocytes and goblet cells is more commonly seen. In humans, this is called “intestinal metaplasia” and is considered to have increased risk for gastric adenocarcinoma [26, 47]. This represents a significant concern when considering the use of TFF3 as a therapeutic molecule.

We report also that short term administration of recombinant TFF3 protein provide the same improvement of glucose tolerance without apparent intestinal metaplasia. It is unclear at this time whether the method of delivery, AAV-mediated liver expression versus IP protein injection, or the duration of exposure is responsible for this difference.

Given its prominent role in cell migration, TFF3 was investigated as a potential wound healing factor to repair the gut epithelium damage observed during the progression of ulcerative colitis. Recombinant TFF3 protein has been delivered in humans via enema in combination with oral 5-aminosalicylic acid (5-ASA) for the treatment of mild-to-moderate left-sided ulcerative colitis [15]. No safety concerns were observed in this clinical trial, however the treatment was discontinued because of failure to find a significant therapeutic effect. The 5-ASA treatment may have masked the beneficial effects of TFF3, or alternatively, longer exposure may have been needed to observe an effect. Oral administration of TFF3 expressing Lactococcus lactis successfully prevented and healed acute colitis in mice [14], which opens the possibility to achieve exposure levels sufficient to treat metabolic or inflammatory bowel diseases in humans. Further studies will be needed to define a potential therapeutic window of TFF3 administration. Alternatively, the identification of TFF3 receptor(s) and signaling pathway(s) could provide additional molecular targets differentiating the neoplasic from the metabolic phenotypes.

## Supporting Information

S1 Raw Data (XLSX)
